# Hypothalamic tanycytes internalize ghrelin from the cerebrospinal fluid: Molecular mechanisms and functional implications

**DOI:** 10.1016/j.molmet.2024.102046

**Published:** 2024-10-12

**Authors:** Ivana M. Gomez, Maia Uriarte, Gimena Fernandez, Franco Barrile, Daniel Castrogiovanni, Sonia Cantel, Jean-Alain Fehrentz, Pablo N. De Francesco, Mario Perello

**Affiliations:** 1 Laboratory of Neurophysiology of the Multidisciplinary Institute of Cell Biology [IMBICE, Argentine Research Council (CONICET) and Scientific Research Commission, Province of Buenos Aires (CIC-PBA), National University of La Plata], La Plata, Buenos Aires, Argentina; 2 Institut des Biomolécules Max Mousseron-UMR5247, Pôle Chimie Balard Recherche, Montpellier, France; 3 Department of Surgical Sciences, Functional Pharmacology and Neuroscience, University of Uppsala, Uppsala, Sweden

**Keywords:** Blood-cerebro spinal barrier, Hypothalamus, Locomotor activity

## Abstract

**Objective:**

The peptide hormone ghrelin exerts potent effects in the brain, where its receptor is highly expressed. Here, we investigated the role of hypothalamic tanycytes in transporting ghrelin across the blood-cerebrospinal fluid (CSF) interface.

**Methods:**

We investigated the internalization and transport of fluorescent ghrelin (Fr-ghrelin) in primary cultures of rat hypothalamic tanycytes, mouse hypothalamic explants, and mice. We also tested the impact of inhibiting clathrin-mediated endocytosis of ghrelin in the brain ventricular system on the orexigenic and locomotor effects of the hormone.

**Results:**

*In vitro*, we found that Fr-ghrelin is selectively and rapidly internalized at the soma of tanycytes, via a GHSR-independent and clathrin-dependent mechanism, and then transported to the endfoot. In hypothalamic explants, we also found that Fr-ghrelin is internalized at the apical pole of tanycytes. In mice, Fr-ghrelin present in the CSF was rapidly internalized by hypothalamic β-type tanycytes in a clathrin-dependent manner, and pharmacological inhibition of clathrin-mediated endocytosis in the brain ventricular system prolonged the ghrelin-induced locomotor effects.

**Conclusions:**

We propose that tanycyte-mediated transport of ghrelin is functionally relevant, as it may contribute to reduce the concentration of this peptide hormone in the CSF and consequently shortens the duration of its central effects.

## Highlights

• Tanycytes internalize ghrelin via a GHSR-independent and clathrin-dependent mechanism.
• Tanycytes internalize ghrelin at the soma and transport it to the endfoot.
• In hypothalamic explants, tanycytes internalize ghrelin at the apical pole.
• Hypothalamic tanycytes internalize CSF ghrelin in a clathrin-dependent manner in vivo.
• Clathrin inhibition in the brain ventricles prolongs locomotor effects of ghrelin.

## 1 Introduction

Plasma contains a diverse array of peptide hormones that play crucial roles in regulating body homeostasis by acting within the central nervous system. However, these peptide hormones do not freely permeate the central nervous system [1]. Instead, their ability to target specific neuronal types depends on a complex interplay of transport mechanisms within intricate anatomical interfaces [2]. Investigating how peptide hormones access the central nervous system holds significant importance, not only for comprehending the physiological actions of these hormones but also for shedding light on their potential use as medications or for addressing fundamental questions related to pathological states, such as the reduction of the central actions of hormones like ghrelin, leptin, or insulin observed in obesity [3]. Despite extensive research in the past decades, certain key aspects regulating the transport of some hormones through the brain-blood interfaces, as is the case with ghrelin, remain unclear or controversial [4,5].

Ghrelin is an acylated peptide hormone predominantly secreted from the stomach that acts through its sole known receptor, the growth hormone secretagogue receptor (GHSR). Since ghrelin is not produced in the central nervous system [6] and GHSR is primarily expressed in the brain, circulating ghrelin needs to access into the brain in order to mediate its potent effects on appetite, neuroendocrine axes activity, reward-related behaviors, mood, and memory, among various other functions [7]. Notably, studies in mice showed that the accessibility of plasma ghrelin to the brain is remarkably low and confined to specific brain areas [5]. In particular, seminal studies involving systemic injections of radioactive mouse ghrelin demonstrated a very low transport rate of this peptide into the mouse brain [8]. Notably, the transport of ghrelin into the brain was unaffected in mice lacking GHSR [9]. Studies using systemic injection of fluorescent variants of ghrelin also found that these peptide analogs fail to reach deep brain regions but instead rapidly and freely extravasate from fenestrated capillaries in specific brain regions, such as the median eminence (ME), the area postrema, and other circumventricular organs [[10], [11], [12], [13], [14], [15]]. The use of fluorescent variants of ghrelin also revealed that centrally and systemically-injected ghrelin analogs are internalized in hypothalamic tanycytes and choroid plexus epithelial cells, which are integral components of the blood-cerebrospinal (CSF) barrier [[16], [17], [18]] A putative role of the blood-CSF barrier mediating the central actions of ghrelin is further supported by the observation that systemically-injected ghrelin reaches the CSF within minutes after injection in sheep and mice [18,19]. From the CSF, ghrelin rapidly diffuses to the brain parenchyma and acts on targets located in close apposition to the brain ventricular system, such as the hypothalamic paraventricular nucleus [18,20]. Finally, immunoblockade of ghrelin in the CSF impairs the delayed effects of ghrelin on food intake [18]. Thus, rapid access of ghrelin through the fenestrated capillaries as well as its transport across the blood-CSF barrier appears to mainly contribute to the central actions of this hormone.

Tanycytes have emerged as key players controlling the transport of certain hormones and metabolites through the blood-CSF barrier. Hypothalamic tanycytes are specialized bipolar glial cells that have their somas along the walls of the third ventricle and a long basal process projecting into the hypothalamic parenchyma (α type) or toward portal vessels in the ME (β type). The somas of tanycytes are connected by tight junctions, impeding the paracellular transport of molecules, and express the machinery for endocytosis/transcytosis/exocytosis [21]. Not without controversies [22,23], some studies indicate that hypothalamic tanycytes selectively transport blood-borne peptide hormones, such as leptin and insulin, or the glucagon-like peptide 1 receptor agonist liraglutide into the CSF [[24], [25], [26], [27]]. In the case of ghrelin, previous studies have shown that hypothalamic tanycytes of mice rapidly internalize centrally or systemically injected fluorescent ghrelin analogs [[16], [17], [18],^28^]. *In vitro*, rat and mouse hypothalamic tanycytes also rapidly internalize fluorescent ghrelin analogs [16,17]. Strikingly, however, hypothalamic tanycytes do not express detectable levels of GHSR, and they are able to internalize fluorescent ghrelin in primary cultures derived from either wild-type (WT) or GHSR-deficient mice [17]. The apparent lack of specificity of hypothalamic tanycytes in internalizing ghrelin has raised concerns regarding their physiological role in transporting this hormone into the brain, particularly in light of the absence of evidence indicating that they indeed can internalize ghrelin at their endfeet and transport to the ventricular compartment.

Here, we report the use of different *in vitro, ex vivo*, and *in vivo* experimental approaches to test the hypothesis that hypothalamic tanycytes can transport plasma ghrelin into the brain. Contrary to our expectations, our results indicate that tanycytes internalize ghrelin from its apical pole, in a clathrin-dependent manner, and transport it to their terminal pole, suggesting that these cells remove ghrelin from the CSF and, consequently, contribute to shortening the duration of its central effects.

## 2 Materials and methods

### 2.1 Animals

Male adult (8–10 weeks old) C57BL/6 wild type mice and Sprague-Dawley rats were housed on a 12-h light/dark cycle with regular chow and water available *ad libitum* unless otherwise specified. This study was carried out in strict accordance with the recommendations in the Guide for the Care and Use of Laboratory Animals of the National Institutes of Health, and all efforts were made to minimize suffering. All experimentation received approval from the Institutional Animal Care and Use Committee of the Multidisciplinary Institute of Cell Biology.

### 2.2 Reagents

Ghrelin (GSS(octanoyl)FLSPEHQKAQQRKESKKPPAKLQPR, 3.3 kDa) and Desacyl-ghrelin (GSSFLSPEHQKAQQRKESKKPPAKLQPR, 3.2 kDa) were purchased from Global Peptide (cat no. PI-G-03 and PI-G-04 respectively). Far-red ghrelin, hereafter named Fr-ghrelin, (GSD(octanoyl)FLSPEHQRVQQRKESC-(DY-647P1)amide, 3.6 kDa) is a 19-amino acid ghrelin analog conjugated to the fluorophore DY-647P1 through a C-terminal Cys. Fr-ghrelin was synthesized as previously detailed [17]. F-ghrelin (GSDpr(octanoyl)FLSPEHQRVQQRKESK(fluorescein)amide, 2.7 kDa) is a 19-amino acid ghrelin analog conjugated to fluorescein isothiocyanate through the side-chain amine of the C-terminal lysine. F-ghrelin was synthesized as previously detailed [29]. Scrambled F-ghrelin, hereafter named scr-F-ghrelin (FRVESKESPQGRDpr(octanoyl)QHSQLK(fluorescein)amide, 2.7 kDa), consists of the identical amino acid residues as F-ghrelin but in a different order. Scr-F-ghrelin was synthesized as previously detailed [18,29]. Pitstop 2 and Dyngo-4a are clathrin-mediated endocytosis inhibitors. Pitstop 2 (Sigma-Aldrich, cat no. SML 1169) inhibits functional interaction sites on the N-terminal domain of clathrin heavy chain, interfering with the clathrin-coated vesicle assembly [30]. Dyngo-4a (Abcam, cat no. ab120689) inhibits dynamin action, preventing membrane fission during clathrin-mediated endocytosis [31,32]. Colchicine (Sigma- Aldrich, cat no. C9754), inhibits microtubule polymerization, thus blocking intracellular transport [33]. JMV2959, a GHSR antagonist, was synthesized as previously detailed [34]. Bis-benzimide (Hoeschst 33258, cat. B1155) and hydroxystilbamidine bis(methanesulfonate) (Fluorogold, cat 39286) were purchased from Sigma-Aldrich. Carboxylate-modified green fluorescent polystyrene microspheres were purchased from ThermoFisher Scientific (FluoSpheres™ cat. F8803). Thrombin was purchased from Sigma (cat. T7009-1KU).

### 2.3 Image acquisition

Fluorescence microscopy images were acquired using a Zeiss AxioObserver D1 microscope equipped with an Apotome.2 structured illumination module, an AxioCam 506 monochrome camera, 10 × /0.45, 20 × /0.8, 40 × /0.95 and 63 × /1.4 (oil) objectives with according DIC prisms and blue, green, red and far-red filter sets (Zeiss 38 HE, 43 HE, 49 and 50, respectively). All images used for fluorescence quantification were acquired under the same ilumination intensity and exposure time for each experimental set.

### 2.4 Primary culture of hypothalamic tanycytes

Hypothalamic tanycytes from P10 Sprague-Dawley rats were cultured as previously described [35]. Briefly, the hypothalamic ME region was dissected from 10-day-old rats, washed three times with Hank's Balanced Salt Solution (HBSS) and cells were dissociated. Cell number and viability were assessed and cells were resuspended in serum-free chemically defined culture medium, plated in 22 × 22 mm cover glasses (100,000–120,000 cells) and incubated at 37 °C with 5% CO2. On day 3, half of the medium was replaced with fresh one, plus the addition of thrombin at a final concentration of 1.25 UI/ml to favor tanycytes growth. Cells were cultured for a total period of 7 days.

### 2.5 Assessment of GHSR mRNA levels in cultured tanycytes

Cultured cells were used to isolate RNA for quantitative RT-PCR. RT-PCR was carried out on Applied Biosystems 7900HT Fast Real-Time PCR System using exon-boundary-specific TaqMan® Gene Expression Assays (Applied Biosystems, cat. 4448488) and RNA isolated from rat hypothalamus as positive control. Oligonucleotide primers and fluorogenic probe sets for TaqMan real-time PCR were manufactured by Applied Biosystems (rat GHSR: Rn00821417; rat actin: Rn00667869).

### 2.6 *In vitro* assessment of Fr-ghrelin internalization in cultured tanycytes

First, cultured tanycytes were washed three times with HBSS and incubated for 5 or 30 min at 37 °C with vehicle (HBSS) alone or containing 300 nM of Fr-ghrelin before being fixed. The cell fixation and mounting procedure was the same in all *in vitro* experiments: after washing 3 times with cold PBS, cells were fixed with paraformaldehyde 4% in PBS at 4 C for 30 min. Cover glasses were mounted using an anti-fading solution containing 10 mg/ml Hoechst to stain cell nuclei. For the cellular mechanism and selectivity assessment, Fr-ghrelin was co-incubated with JMV2959, ghrelin, desacyl-ghrelin, scr-F-ghrelin, Pitstop 2 or Dyngo-4a. For this purpose, cells were washed three times with HBSS and pre-incubated for 2 min at 37 °C with 3 μM JMV2959, 300 nM ghrelin, 300 nM desacyl-ghrelin, 300 nM scr-F-ghrelin, 30 μM Pitstop 2 or 30 μM Dyngo-4a. Afterwards, the incubation solution was replaced with culture media of the same composition plus the addition of 300 nM Fr-ghrelin and incubated for other 5 min at 37 °C. Finally, cells were fixed and coverslipped. Fr-ghrelin internalization in tanycytes was determined by fluorescence microscopy, as described above. Quantification of fluorescence intensity in the soma, process, and endfoot of individual tanycytes was performed using a custom-made macro in Fiji [36]. Relative mean fluorescence was calculated by normalizing the mean fluorescence intensity measurements to the average mean fluorescence intensity of the positive control of each independent experiment (cells incubated with Fr-ghrelin only) and multiplying by 100. As a complementary maneuver to study the mechanism of ghrelin internalization in hypothalamic tanycytes, 5-min co-incubations at 37 °C with a solution containing 300 nM Fr-ghrelin and 2 μg/ml fluorescent microspheres were performed. Finally, cells were washed with cold PBS, fixed, coverslipped and imaged.

### 2.7 Immunocytochemical studies

Cultured tanycytes were washed and incubated at 37 °C with HBSS alone or containing 300 nM Fr-ghrelin or 300 nM ghrelin for 5 min, as described above. After fixation, cells were treated with blocking solution (3% normal donkey serum and 0.25% Triton X-100 in PBS). Cells pre-incubated with ghrelin were incubated with goat anti-ghrelin antibody (Santa Cruz, cat. sc-10368, 1:500), whereas cells pre-incubated with Fr-ghrelin were incubated either with anti-clathrin (Cell Signaling, cat. 2410, 1:1000), anti-rab5 (Cell Signaling, cat. 3547, 1:1000) or anti-transferrin receptor (TfR, Invitrogen, cat. 13–6800, 1:500) antibodies made in rabbit, in blocking solution for 24 h at 4 °C. Next, slides were washed three times with PBS and incubated for 1 h at room temperature with either donkey anti-goat IgG conjugated to Alexa 594, or goat anti-rabbit IgG conjugated to Alexa 488 (Invitrogen, cat. A11058 and A11008, respectively, 1:1000), as appropriate. Finally, slides were coverslipped and fluorescence images were acquired, as described above.

### *2.8* *In vitro* study of Fr-ghrelin transport

Co-incubations with colchicine and Fr-ghrelin were performed by first incubating cells at 37 °C with 1 mM colchicine for 2 min and then with a solution containing 1 mM colchicine and 300 nM Fr-ghrelin for 5 min. Finally, cells were washed with cold PBS, fixed and coverslipped as mentioned before. For the pulse-chase experiments, cultured tanycytes were washed three times and a 5-min incubation with 300 nM Fr-ghrelin was performed. Then, cells were rewashed and incubated with culture medium at 37 °C with 5% CO_2_ for 10 or 25 min. After this, cultured tanycytes were washed with cold PBS, fixed and coverslipped. Fr-ghrelin internalization in the soma, process and endfoot of individual tanycytes was assessed by fluorescence microscopy imaging followed by analysis in Fiji, as detailed above.

### *2.9* *Ex vivo* imaging studies in hypothalamic explants

To obtain hypothalamic explants, WT mice were euthanized by decapitation and their brains removed from the skull. Coronal sectioning was performed using a brain blocker to obtain 3 mm thick slices containing the entire hypothalamus. Each slice was then placed in a Petri dish containing cold HBSS. Using a stereomicroscope, a horizontal section was made to obtain a block of tissue containing the entire dorsal part of the third ventricle. At this point, two different manipulations were performed: incubations with tracers either inside the third ventricle or on the outer surface of the explants. In the first case, 2 μl of 1% Fluorogold or 30 μM Fr-ghrelin was injected into explants within the third ventricle using a stereomicroscope and a micromanipulator. The explants were then placed, ventral side down, in a custom-made incubation chamber containing a cavity that allows the outer side of the hypothalamus to rest immersed in an incubation solution, in this case HBSS. In the second case, once the explants were obtained, they were placed directly in the incubation chamber in the same spatial orientation as mentioned before. Thus, the outer part of the hypothalamus was completely immersed in 50 μl of 0.1% Fluorogold or 2.2 μM Fr-ghrelin, as appropriate. In both cases, the custom-made chamber containing the explant was placed in a humid chamber at 37 C and the explants were incubated for 15 min. Tissue was washed in HBSS, fixed in 4% formaldehyde overnight and then immersed in 20% sucrose overnight. Finally, the explants were frozen and cut into four equal series of 40-μm coronal sections on a sliding cryostat. The fluorescent signal of Fr-ghrelin in tanycytes was observed by direct fluorescence microscopy.

### *2.10* *In vivo* imaging studies in mice

Fr-ghrelin was administered to WT mice by acute intracerebroventricular (ICV) injections. For this purpose, mice were stereotaxically implanted into the lateral ventricle with an indwelling guide cannula (length 4 mm, 22-gauge). The placement coordinates were: anteroposterior: −0.3 mm; lateral: +1.0 mm and ventral: −2.3 mm. At this anteroposterior level, lateral ventricles are connected to the dorsal part of the third ventricle and ICV-injected molecules quickly reach it. Mice were ICV-injected with 2 μL of artificial CSF (aCSF: 140 mM NaCl, 3.35 mM KCl, 1.15 mM MgCl_2_, 1.26 mM CaCl_2_, 1.2 mM Na_2_HPO_4_, and 0.3 mM NaH_2_PO_4_, pH 7.4 in sterile water) alone or containing 60 pmol/mouse of Fr-ghrelin, as previously detailed [18]. Mice were anesthetized and perfused after 15, 30, 60, or 90 min with 4% formaldehyde, as described in the past [37]. To assess the selectivity of ghrelin internalization, a solution containing 60 pmol/mouse of Fr-ghrelin and 60 pmol/mouse of Scr-F-ghrelin or 50 μg/mouse of green fluorescent microspheres was similarly injected into another group of mice that were perfused after 15 min. Pitstop 2 was also administered to a different group of mice prior to ICV administration of F-ghrelin to study the cellular mechanism of ghrelin uptake in tanycytes. In this case, mice were stereotaxically implanted with a single indwelling ICV guide cannula as described above, housed individually, and allowed to recover for at least 5 days. On the morning of the experimental day, mice were ICV-injected with aCSF alone or containing 6 nmol/mouse of Pitstop 2, and 1 h later, were anesthetized and ICV-injected with 60 pmol/mouse of F-ghrelin. The animals were perfused after 15 min. In every case, brains from perfused mice were postfixed, immersed in 20% sucrose, frozen, and coronally sectioned at 40 μm in four equal series on a sliding cryostat. Correct placement of the cannula was confirmed by histologic observation at the end of the experiment. Fluorescence images were acquired as detailed above and microphotographs were analyzed using a custom-made macro in Fiji. In short, a ROI comprising the somas of tanycytes and another ROI comprising the rest of the ME tissue containing the processes and terminals of tanycytes, hereafter called “external ME”, were determined. Mean fluorescence intensity was measured in both ROIs.

### 2.11 Behavioral assessments

WT mice were stereotaxically implanted with an ICV guide cannula in the lateral ventricle as described before, housed individually and allowed to recover for at least 5 days before the experiments. Animals were then used to assess locomotor activity and food intake in response to ICV treatment, divided into 4 experimental groups that received two ICV injections per day on consecutive days. For the locomotor activity assessment, injections were made in a crossover design fashion. The experimental groups, here referred to as “injection 1 + injection 2″, were: 1) vehicle + vehicle, 2) Pitstop 2 + vehicle, 3) vehicle + ghrelin and 4) Pitstop 2 + ghrelin. Mice were euthanized at the end of the procedure and their brains were removed to confirm the correct placement of the cannula by histologic observation. During the post-surgery recovery period, mice were habituated to handling by removing the dummy cannula and connecting it to an empty cannula connector. On the experimental day, mice were ICV-injected using a 30-gauge needle, as previously described [38], with 2 μL of vehicle alone or containing 6 nmol/mouse of Pitstop 2. After 5 min, mice received a second ICV injection of vehicle or 300 pmol/mouse of ghrelin, as appropriate.

Locomotor activity was registered, as previously described in detail [38], in recording cages placed in a ventilated and acoustically isolated monitoring box equipped with an overhead camera and dimmable LED lighting. Prior to assessment, the mice were habituated to the recording environment by transferring each mouse from its home cage to its dedicated recording cage, which in turn was placed in the monitoring box for 1 h on 3 consecutive days. On the experimental day, mice received the two ICV injections mentioned above and were then transferred to the recording cage and placed in the monitoring box. Locomotor activity was recorded for 60 min after treatment, videos were imported into Fiji and processed using custom-made macro to extract the total distance traveled for each mouse, expressed in centimeters.

Food intake was determined as previously described [38]. Briefly, all food pellets were removed from the home cage hoppers and the bedding was confirmed to be free of chow remains. After the mice were removed from the recording cages, they were returned to their living cages where they were exposed to a single pre-weighed chow pellet (∼1500 mg). Mice remained undisturbed for 2 h, while chow pellets and any additional chow spillage were collected and weighed. 30-min, 1-h and 2-h food intake was calculated by subtracting the remaining weight of the pellet from the initial weight, and expressed in mg.

### 2.12 Determination of CSF ghrelin levels

WT mice were stereotaxically implanted with an ICV guide cannula in the lateral ventricle, housed individually and allowed to recover for at least 5 days before the experiments. On the experimental day, mice were ICV-injected with 2 μL of vehicle alone or containing 6 nmol/mouse of Pitstop 2, as appropriate. After 5 min, mice received a second ICV injection of 300 pmol/mouse of ghrelin. 45 min later, 10–15 μL of CSF was collected from the cisterna magna as described elsewhere [39]. Briefly, mice were anesthetized and placed in a stereotaxic frame with their head forming a nearly 135° angle with respect to the body. Under a binocular lens, an incision inferior to the occiput was made and the subcutaneous tissue and muscles were separated by blunt dissection with forceps. A 100-μm-point capillary tube was inserted into the cisterna magna through the dura mater, allowing the CSF to flow into it. CSF samples were immediately frozen on dry ice for later storage at −80 °C. Mice were euthanized at the end of the procedure and their brains were removed. Correct placement of the cannula was confirmed by histologic observation. Ghrelin concentration was assayed in the collected CSF samples using a specific acylated ghrelin ELISA kit (Bertin Pharma, cat no. A05117, intra-assay variation 8.1%, detection limit 6.7 pg/mL) following manufacturer's recommendations.

### 2.13 Statistical analyses

Normality and homogeneity of variances were tested using the D'Agostino & Pearson omnibus test or Bartlett's test, respectively. When a normal distribution was found, data were expressed as the mean ± standard error of the mean (SEM), and the experimental groups were compared with Student's unpaired t-test, One-way ANOVA or Two-way ANOVA, depending on the number of groups or experimental design. When a normal distribution was not found, data were expressed as median and interquartile range and compared with the Kruskal–Wallis or Mann-Whitney test. Samples sizes, tests used for each comparison and statistical outputs are indicated in the figure legends or the text depending if data summaries are shown as graphs or text, respectively. Differences were considered significant when P < 0.05. All statistical analyses were conducted with the software GraphPad Prism, version 9.0.

## 3 Results

### 3.1 Fr-ghrelin is selectively internalized in cultured hypothalamic tanycytes via a GHSR-independent mechanism

**Figure 1:**
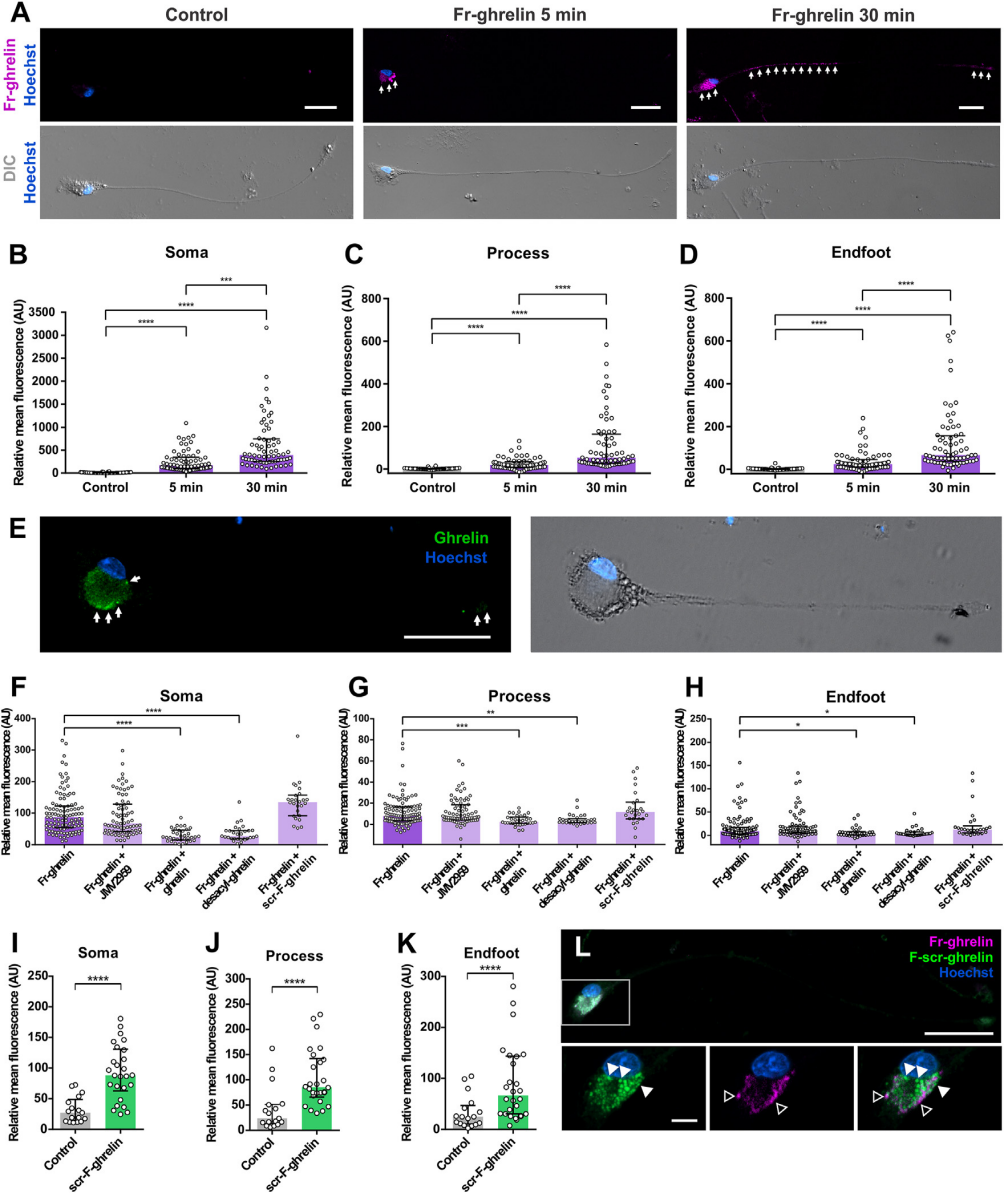
**Fr-ghrelin is selectively internalized in cultured hypothalamic tanycytes via a GHSR-independent mechanism.** Panel **A** shows representative microphotographs of cultured tanycytes either in control conditions or after a 5 or 30-min incubation with Fr-ghrelin (magenta) imaged in fluorescence and DIC. Scale bars: 30 μm, 40 × objective. Panels **B**, **C** and **D** show relative mean fluorescence (AU) in soma, process and endfoot of cultured tanycytes after an incubation with vehicle alone (n = 52 cells from 3 independent experiments) or in the presence of Fr-ghrelin during 5 (n = 68, 4 independent experiments) or 30 min (n = 67, 4 independent experiments). Kruskal Wallis P < 0.0001. Panel **E** shows representative microphotographs of an immunocytochemistry against ghrelin (green) in a cultured tanycyte previously incubated with ghrelin for 5 min and imaged in DIC or fluorescence. Scale bars: 50 μm, 63 × objective. Panels **F**, **G** and **H** show relative mean fluorescence (AU) in soma, process and endfoot of cultured tanycytes after a 5-min incubation with Fr-ghrelin alone (n = 108, 5 independent experiments) or in the presence of JMV2959 (n = 76, 2 independent experiments), ghrelin (n = 34, 2 independent experiments), desacyl-ghrelin (n = 30, 2 independent experiments) or scr-F-ghrelin (n = 26, 2 independent experiments). Kruskal Wallis P < 0.0001. Panels **I**, **J** and **K** show relative mean fluorescence in the channel used for scr-F-ghrelin (AU) in soma, process and endfoot of cultured tanycytes after a 5-min incubation with vehicle alone (n = 17, 1 independent experiment) or in the presence of Fr-ghrelin and scr-F-ghrelin (n = 26, 2 independent experiments). Kruskal Wallis P < 0.0001. In every case, multiple comparisons were made using Dunn's post hoc test. ∗∗∗∗P < 0.0001; ∗∗∗P < 0.001; ∗∗P < 0.01; ∗P < 0.05. Panel **L** shows a representative microphotograph of a cultured tanycyte incubated with Fr-ghrelin (magenta) and scr-F-ghrelin (green) for 5 min. Magnified insets of the soma are presented below. Empty arrowheads indicate Fr-ghrelin and full arrowheads indicate scr-F-ghrelin. Scale bars: 50 μm for 40 × objective and 10 μm for 63 × objective. In every case, cell nuclei were labeled with Hoechst. (For interpretation of the references to colour in this figure legend, the reader is referred to the Web version of this article.)

In order to investigate the mechanisms mediating the internalization of ghrelin in hypothalamic tanycytes, we utilized primary cultures of rat hypothalamic tanycytes, which maintain their *in vivo* morphology [35]. As previously reported [16,17], we found fluorescent signal internalized in rat hypothalamic tanycytes incubated with Fr-ghrelin, as compared to tanycytes incubated with medium alone (Figure 1A). The fluorescent signal within tanycytes was primarily observed in the soma but was also present in the process and endfoot of the cells after either 5 or 30 min of incubation. Levels of fluorescent signal were higher in all cellular compartments after 30 min of incubation with Fr-ghrelin, compared to 5 min of incubation with Fr-ghrelin (Figure 1B–D). To assess if intracellular signal corresponds to intact Fr-ghrelin, we exposed cultured tanycytes to full-length ghrelin and then performed immunostaining against the N-terminal octanoylated end of the peptide. We found ghrelin immunoreactive signal mainly in the somas but also in the processes and endfeet of cells incubated with ghrelin (Figure 1E), as seen in cells incubated with Fr-ghrelin, suggesting that internalized ghrelin remains intact. Next, we assessed the selectivity of the internalization of Fr-ghrelin. Cultured rat tanycytes lack detectable levels of GHSR mRNA (Ct > 40), similarly as reported in hypothalamic mouse tanycytes [17]. To evaluate the selectivity of ghrelin internalization, we assessed if Fr-ghrelin was internalized in cultured tanycytes pre-exposed to saturating concentrations of different GHSR ligands or ghrelin-related peptides (Figure 1F–H). We found that levels of the fluorescent signal in the soma, process, and endfoot of tanycytes were not affected by preincubation with JMV2959, a well-established GHSR blocker. Additionally, the amount of Fr-ghrelin fluorescent signal in the soma, process, and endfoot was not affected by preincubation with scr-F-ghrelin, a peptide containing the same amino acid residues as ghrelin but with a rearranged sequence. This peptide is readily internalized by cultured tanycytes but shows a different intracellular distribution compared to Fr-ghrelin (Figure 1I–L). Conversely, the level of fluorescent signal in the soma, process and endfoot of tanycytes was reduced after the preincubation with full-length ghrelin or desacyl-ghrelin, as compared to the level of fluorescent signal found after preincubation with Fr-ghrelin alone.

### 3.2 Fr-ghrelin is internalized in cultured hypothalamic tanycytes of rats via a clathrin-dependent mechanism

**Figure 2:**
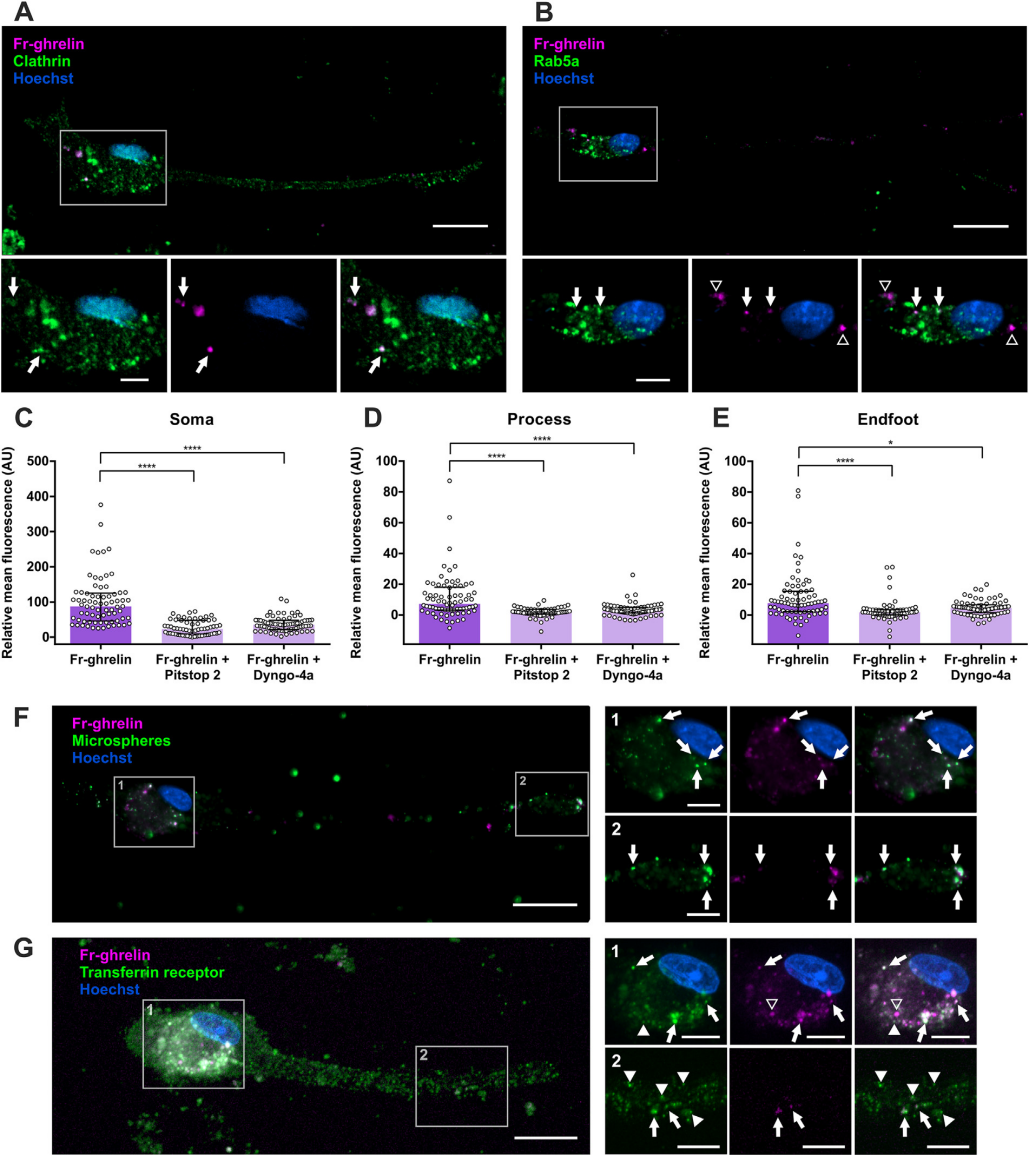
**Fr-ghrelin is internalized via a clatrhin-dependent mechanism in cultured hypothalamic tanycytes**. Panel **A** shows an immunocytochemistry against clathrin (green) in a cultured tanycyte previously incubated with Fr-ghrelin for 5 min (magenta). Magnified insets are presented below, showing the soma of the cell. Arrows indicate clatrhin-IR signal that colocalize with Fr-ghrelin. Scale bars: 20 μm, 63 × objective. Panel **B** shows an immunocytochemistry against Rab5a (green) in a cultured tanycyte previously incubated with Fr-ghrelin for 5 min (magenta). Magnified insets are presented below, showing the soma of the cell. Arrows indicate Rab5a-IR signal that colocalize with Fr-ghrelin and arrowheads indicate Fr-ghrelin that does not colocalize with Rab5a-IR signal. Scale bars: 20 μm, 63 × objective. Panels **C**, **D** and **E** show relative mean fluorescence in soma, process and endfoot of cultured tanycytes after a 5-min incubation with Fr-ghrelin alone (n = 74, 2 independent experiments) or in the presence of Pitstop 2 (n = 73, 2 independent experiments) or Dyngo-4a (n = 61, 2 independent experiments). Kruskal Wallis P < 0.0001. Multiple comparisons were made using Dunn's post hoc test. ∗∗∗∗P < 0.0001; ∗P < 0.05. Panel **F** shows a representative microphotograph of a cultured tanycyte previously incubated with Fr-ghrelin (magenta) and fluorescent microspheres (green) for 5 min. Arrows indicate fluorescent microspheres colocalizing with Fr-ghrelin signal. Scale bars: 30 μm for low or 10 μm for high magnification, 63 × objective. Panel **G** shows an immunocytochemistry against Transferrin Receptor (TfR, green) in a cultured tanycyte previously incubated with Fr-ghrelin for 5 min (magenta). Magnified insets showing the soma and endfoot of the cell are presented. Arrows indicate Fr-ghrelin colocalizing with TfR-IR signal, empty arrowheads indicate Fr-ghrelin that does not colocalize with TfR and full arrowheads indicate TfR-IR signal that does not colocalize with Fr-ghrelin. Scale bars: 30 μm for low or 10 μm for high magnification, 63 × objective. In every case, cell nuclei were labeled with Hoechst. (For interpretation of the references to colour in this figure legend, the reader is referred to the Web version of this article.)

Next, we investigated the cellular mechanisms mediating the internalization of Fr-ghrelin in hypothalamic tanycytes. As active endocytosis is inhibited below 4 °C, we exposed hypothalamic tanycytes to Fr-ghrelin in ice-cold medium and found that the internalization of the fluorescent ghrelin analog was fully inhibited (mean fluorescence intensity per cell: 79.1 ± 67.7 vs 54.8 ± 17.4 AU for 37 °C or 4 °C incubated cells, respectively; Mann Whitney test P = 0.0005). In order to clarify the endocytosis pathway mediating Fr-ghrelin internalization, we performed immunostaining against clathrin in cultured tanycytes pre-incubated with Fr-ghrelin. We found that clathrin immunoreactive signal was present in all compartments of tanycytes, but it mainly colocalizes with Fr-ghrelin in the soma (Figure 2A). Using cultured tanycytes pre-incubated with Fr-ghrelin, we also performed immunostaining against Rab5, a marker of early endosomes that is involved at the beginning of the endocytic pathway, and against TfR, a marker of early as well as recycling endosomes. In contrast to clathrin, Rab5 was highly enriched in the soma of tanycytes, where it partially colocalized with Fr-ghrelin signal (Figure 2B). TfR was found in the somas of tanycytes, as well as in the fibers and endfeet, and partially colocalized with the Fr-ghrelin signal in all three intracellular compartments (Figure 2G). To functionally test the molecular events mediating ghrelin endocytosis, we assessed the internalization of Fr-ghrelin in the presence of Pitstop 2, a cell membrane permeable clathrin inhibitor, or Dyngo-4a, a dynamin inhibitor. We found that the level of fluorescent signal in all three cell compartments of tanycytes was reduced by both Pitstop 2 or Dyngo-4a (Figure 2C–E). In the somas, the level of florescent signal was reduced by 80% and 65% with Pitstop 2 and Dyngo-4a, respectively, compared to the control group. As an additional maneuver to assess the selectivity of the internalization of Fr-ghrelin, we exposed cultured tanycytes to Fr-ghrelin and green fluorescent microspheres. We found that the fluorescent microspheres were present in all three intracellular compartments of tanycytes, where they colocalized with Fr-ghrelin (Figure 2F).

### 3.3 Fr-ghrelin is internalized at the soma of cultured tanycytes and then transported to the endfoot

**Figure 3:**
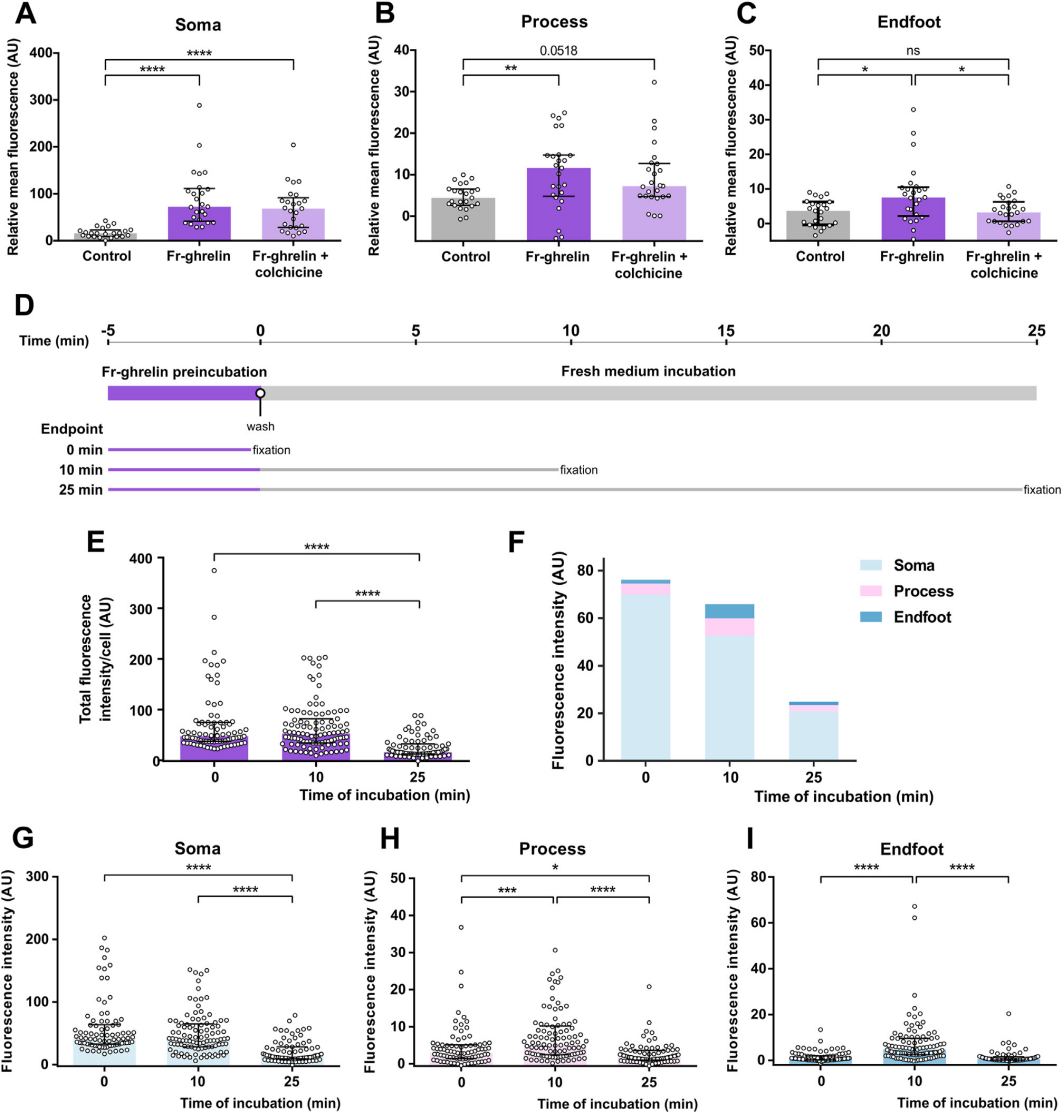
**Fr-ghrelin is mainly internalized at the soma of hypothalamic tanycytes *in vitro***. Panels **A**, **B** and **C** show relative mean fluorescence (AU) in soma, processes and endfeet of cultured tanycytes after a 5-min incubation either with vehicle (n = 23, 2 independent experiments) or Fr-ghrelin alone (n = 25, 2 independent experiments) or in presence of colchicine (n = 25, 2 independent experiments). Kruskal Wallis P < 0.0001 for soma, P = 0.0054 for process and P = 0.0093 for endfoot. Panel **D** shows the experimental outline used in the pulse-chase experiments. After a 5-min incubation with Fr-ghrelin, cells were washed an incubated in fresh medium for 10 or 25 min previous to fixation. Panel **E** shows the total fluorescence intensity per cell (AU) of cultured tanycytes at 0 (n = 83, 3 independent experiments), 10 (n = 101, 3 independent experiments), and 25 min (n = 79, 3 independent experiments) post incubation with Fr-ghrelin. Kruskal Wallis P < 0.0001. Bar graph in panel **f** shows the mean of the fluorescence intensity (AU) for soma, process and endfoot of the cultured tanycytes mentioned in panel e. Panel **G**, **H** and **I** show fluorescence intensity (AU) in soma, process and endfoot of cultured tanycytes at 0, 10 and 25 min post incubation with Fr-ghrelin. Kruskal Wallis P < 0.0001. In every case, multiple comparisons were made using the Dunn's post hoc test. ∗∗∗∗P < 0.0001; ∗∗P < 0.01; ∗P < 0.05.

The simultaneous increase in fluorescent signal in the entire cell body of tanycytes continuously incubated with Fr-ghrelin prevents us from determining whether the fluorescent analog is internalized at a specific cellular region and then internally transported. Since tanycytes are polarized along the soma-endfoot axis, we tested if the internalization of Fr-ghrelin preferentially occurs at one cellular compartment. First, we assessed the fluorescent signal within the tanycytes incubated with Fr-ghrelin in the presence of colchicine, which inhibits tubulin polymerization and blocks intracellular transport. Colchicine treatment did not affect the level of Fr-ghrelin signal in the soma and process compared to tanycytes incubated with Fr-ghrelin alone, but it reduced the Fr-ghrelin signal in the endfoot to the values detected in tanycytes incubated with medium alone (Figure 3A–C). In order to better track the dynamic of Fr-ghrelin within the tanycytes, we performed pulse-chase studies in which tanycytes were preincubated with Fr-ghrelin for 5 min, washed and then incubated with fresh medium to study the presence of Fr-ghrelin within the tanycytes after 0, 10 and 25 min (Figure 3D). We found that total Fr-ghrelin within tanycytes decreased over time (Figure 3E). However, fluorescent signal in each compartment displayed a particular pattern (Figure 3F): Fr-ghrelin signal was reduced at 25 min of incubation in fresh media in the soma (Figure 3G) and process (Figure 3H). Conversely, Fr-ghrelin signal in the endfoot transiently increased, at 10 min of incubation in fresh media, and then decreased at 25 min of incubation in fresh media (Figure 3I).

### 3.4 Fr-ghrelin is internalized at the apical pole of the hypothalamic tanycytes *ex vivo*

**Figure 4:**
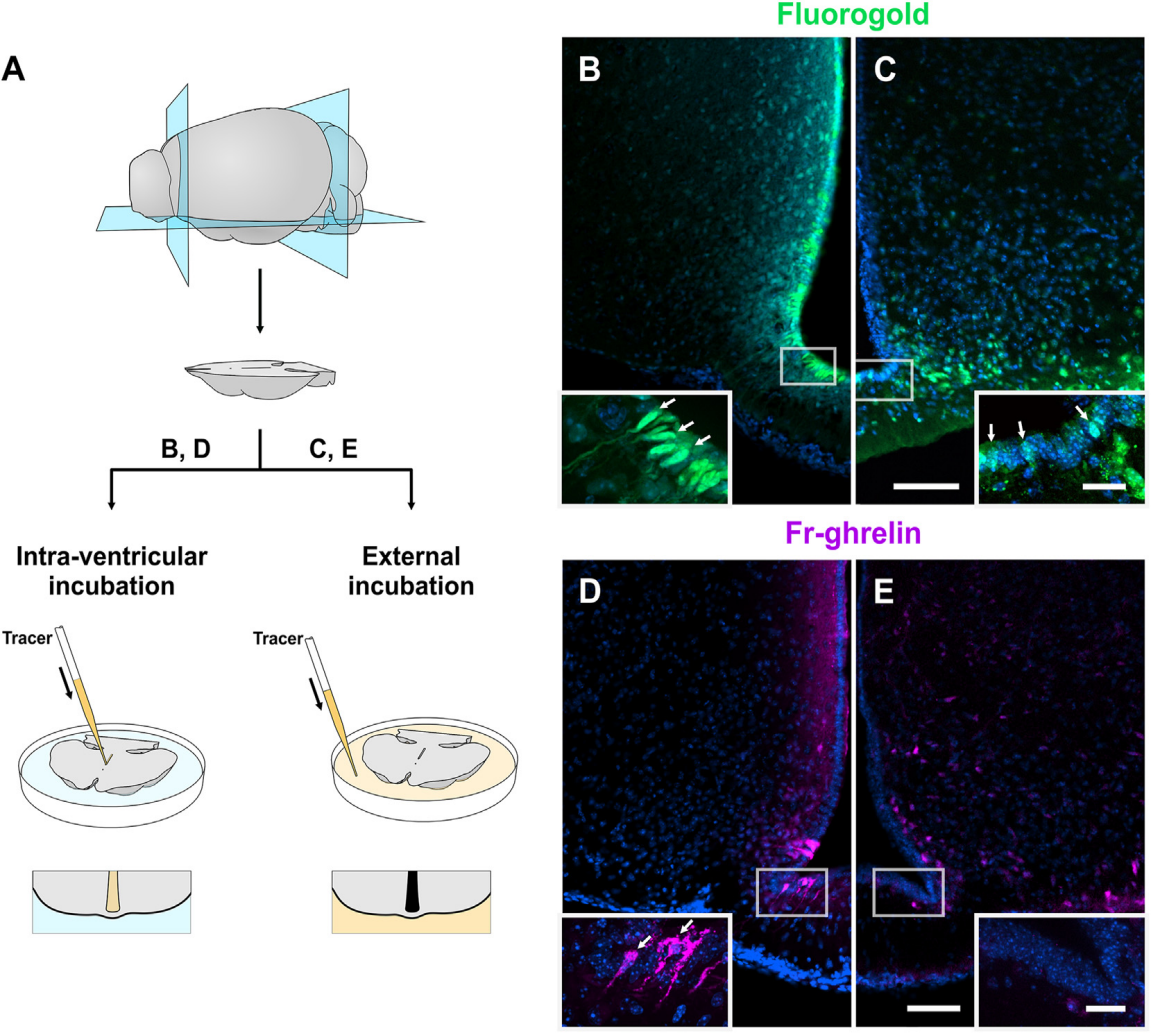
**Fr-ghrelin is mainly internalized at the apical side of the hypothalamic tanycytes *ex vivo***. Panel **a** shows the experimental outline used in *ex vivo* assays. Hypothalamus explants were obtained and incubated with Fluorogold or Fr-ghrelin, either within the ventricle or in contact with its external region. Panels **B** and **C** show representative micrographs of coronal sections of explants incubated with Fluorogold (green) inside the ventricle or on their external side, respectively. Magnified insets are presented below. Arrows indicate fluorescent signal corresponding to Fluorogold in the somas of tanycytes. Panels **D** and **E** show representative micrographs of coronal sections of explants incubated with Fr-ghrelin (magenta) inside the ventricle or on their external region, respectively. Magnified insets are presented below. Arrows indicate fluorescent signal corresponding to Fr-ghrelin in the somas of tanycytes in d. In both cases, cell nuclei were labeled with Hoechst. Scale bars: 100 μm for low magnification (10 × objective) or 30 μm for high magnification (40 × objective). (For interpretation of the references to colour in this figure legend, the reader is referred to the Web version of this article.)

In order to independently assess the capability of the apical or terminal pole of the tanycytes to internalize Fr-ghrelin in a conserved structural context, we developed an experimental strategy in which horizontally cut ventral blocks of the mouse hypothalamus are incubated *ex vivo* in a two-compartment chamber, maintaining the intra-ventricular surface compartmentalized form the external pial surface (Figure 4A). The system allows the independent manipulation of either the inner compartment comprising the volume of the ventral third ventricle, where the apical poles of the tanycytes are located, or the outer compartment that is in contact with the external region of the ME, where the processes and endfeet of tanycytes project. First, we tested the validity of the experimental model by adding Fluorogold to either the inner or the outer compartments, and we confirmed that tanycytes can internalize the fluorescent tracer from both sides (Figure 4B–C). Next, we added Fr-ghrelin in the inner compartment and found fluorescent signal as a diffuse pattern in the periventricular hypothalamic tissue, in close apposition to the ventricular wall, as well as the entire cell body of the β tanycytes (Figure 4D). When Fr-ghrelin was added in the outer compartment, fluorescent signal was found in cells located in the ventromedial region of the ARH whereas no evident signal was observed in tanycytes (Figure 4E).

### 3.5 Fr-ghrelin present in the CSF is rapidly internalized in hypothalamic tanycytes *in vivo*, in a similar fashion as seen *in vitro*

**Figure 5:**
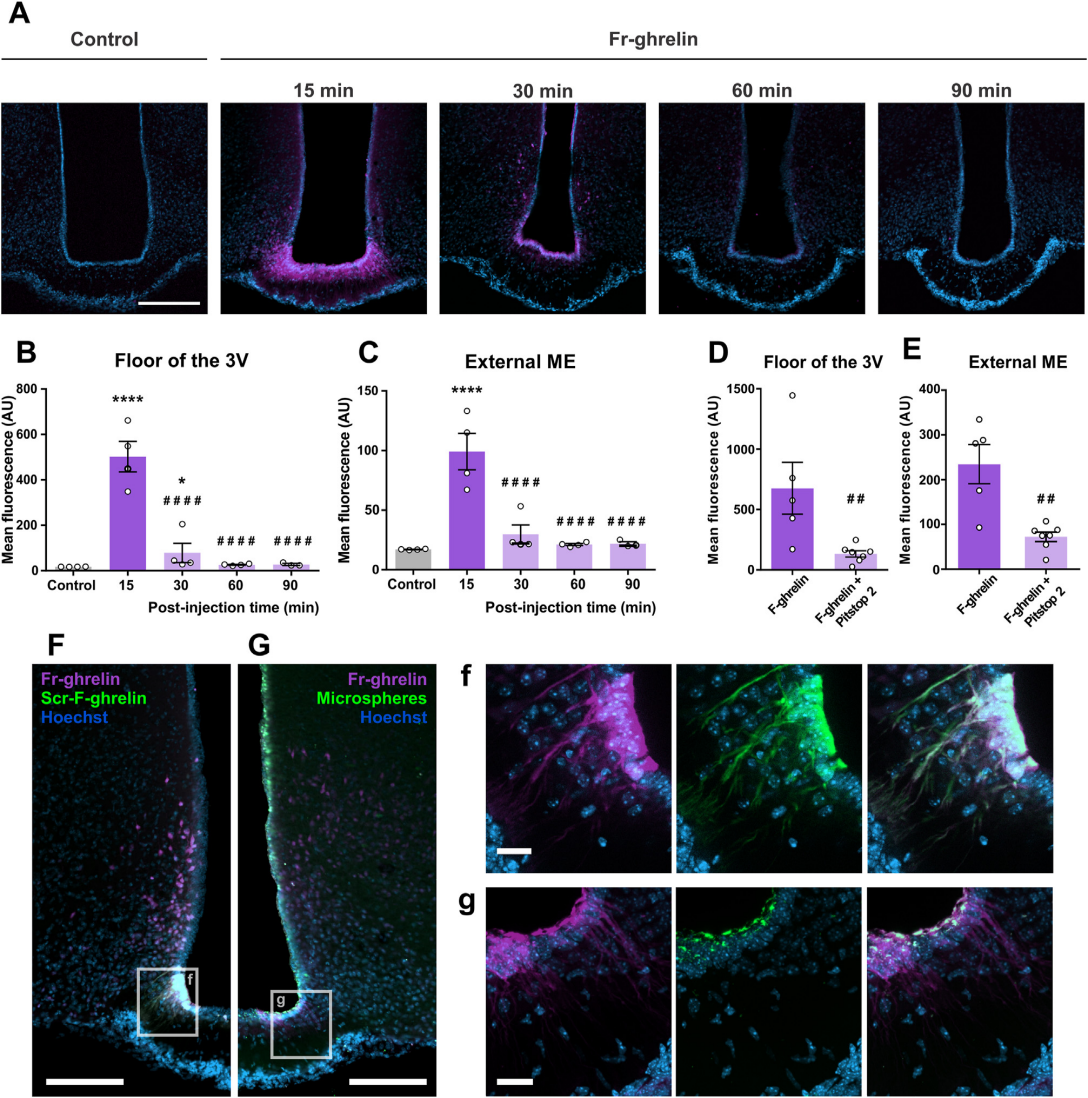
**Fr-ghrelin present in the CSF is rapidly internalized in hypothalamic tanycytes *in vivo*, in a similar fashion as seen *in vitro***. Panel **A** shows representative microphotographs of the median eminence of mice centrally injected either with vehicle (control) or Fr-ghrelin and perfused after 15, 30, 60 or 90 min. Scale bar: 200 μm (10 × objective). Panels **B** and **C** show the mean fluorescence intensity (AU) either in the floor of the 3V or the external ME of mice belonging to the experimental groups mentioned above (control conditions: n = 4; Fr-ghrelin 15 min: n = 4, 30 min: n = 4, 60 min: n = 4, 90 min: n = 3). One-way ANOVA of the logarithm of mean fluorescence intensity (F_floor of the 3V_ (4, 14) = 34.53; P < 0.0001; F_external ME_ (4, 15) = 14.33; P < 0.0001). In every case, multiple comparisons were made using Sidak's post hoc test. For the floor of the 3V: ∗∗∗∗P < 0.0001 vs vehicle; ∗P < 0.05 vs vehicle; ####P < 0.0001 vs Fr-ghrelin 15 min. For external ME: ∗∗∗∗P < 0.0001 vs vehicle; ###P < 0.001 vs Fr-ghrelin 15 min; ##P < 0.01 vs Fr-ghrelin 15 min. Panel **D** and **E** show the mean fluorescence intensity (AU) in the floor of the 3V or the external ME of mice ICV injected with F-ghrelin alone (n = 5) or in the presence of Pitstop 2 (n = 7). Unpaired t-test of the logarithm of mean fluorescence intensity P_floor of the 3V_ = 0.0048; P_external ME_ = 0.0037. Panel **F** shows representative microphotographs of the 3V wall of mice centrally injected with Scr-F-ghrelin (green) and Fr-ghrelin (magenta). Magnified insets are presented in **f**, showing Scr-F-ghrelin and Fr-ghrelin signal colocalizing in β-tanycytes. Panel **G** shows representative microphotographs of the 3V wall of mice centrally injected with fluorescent microspheres (green) and Fr-ghrelin (magenta). Magnified insets are presented in **g**, showing microspheres and Fr-ghrelin signal colocalizing in the soma of β-tanycytes. Scale bars: 200 μm for low magnification (10 × objective) and 30 μm for high magnification (63 × objective). In every case, cell nuclei were labeled with Hoechst. (For interpretation of the references to colour in this figure legend, the reader is referred to the Web version of this article.)

To investigate the kinetics of ghrelin internalization *in vivo*, we centrally injected Fr-ghrelin in mice and assessed the presence of the tracer in hypothalamic tanycytes at different times after injection. As shown in the past [17], centrally injected Fr-ghrelin was mainly found in β tanycytes and in some α tanycytes (Figure 5A). Notably, the level of fluorescent signal transiently increased in both the floor of the third ventricle, where somas of tanycytes are located, and in the external zone of the ME, where the processes and endfeet of the tanycytes are located (Figure 5A–C). The fluorescent signal reached its maximum at 15 min post-injection of Fr-ghrelin in both regions of the ME, and then it was reduced to control levels at 60- and 30-min post-injection, in the floor of the third ventricle and the external zone of the ME, respectively (Figure 5A–C). Next, we studied if Pitstop 2 pre-treatment affected the internalization of fluorescent ghrelin in hypothalamic tanycytes of mice centrally injected with the tracer. We found that the fluorescent signal in both the floor of the third ventricle and the external ME was reduced by 85% and 100%, in mice pretreated with Pitstop 2 (Figure 5D–E). Of note, fluorescent signal was also reduced in the epithelial cells of the choroid plexus of mice pretreated with Pitstop 2 (log of mean fluorescence intensity: 2.5 ± 0.2 vs 2.0 ± 0.1 AU for mice pretreated with vehicle or Pitstop 2, respectively; unpaired t-test P = 0.045). To assess the selectivity of β tanycytes to internalize Fr-ghrelin, we centrally injected mice with Fr-ghrelin together with either scr-F-ghrelin or fluorescent microspheres. In mice co-injected with Fr-ghrelin and scr-F-ghrelin, scr-F-ghrelin was exclusively found in β tanycytes that were also labeled by Fr-ghrelin (Figure 5F,f). In mice co-injected with Fr-ghrelin and fluorescent microspheres, the fluorescent microspheres were found in all cells of the ependymal layer of the third ventricle, including β tanycytes that were also labeled by Fr-ghrelin (Figure 5G,g). Thus, centrally injected Fr-ghrelin appears to be rapidly internalized, via a clathrin-dependent mechanism, in hypothalamic tanycytes that are also capable of internalizing other cargo molecules present in the CSF.

### 3.6 The internalization of ghrelin from the CSF has functional implications

**Figure 6:**
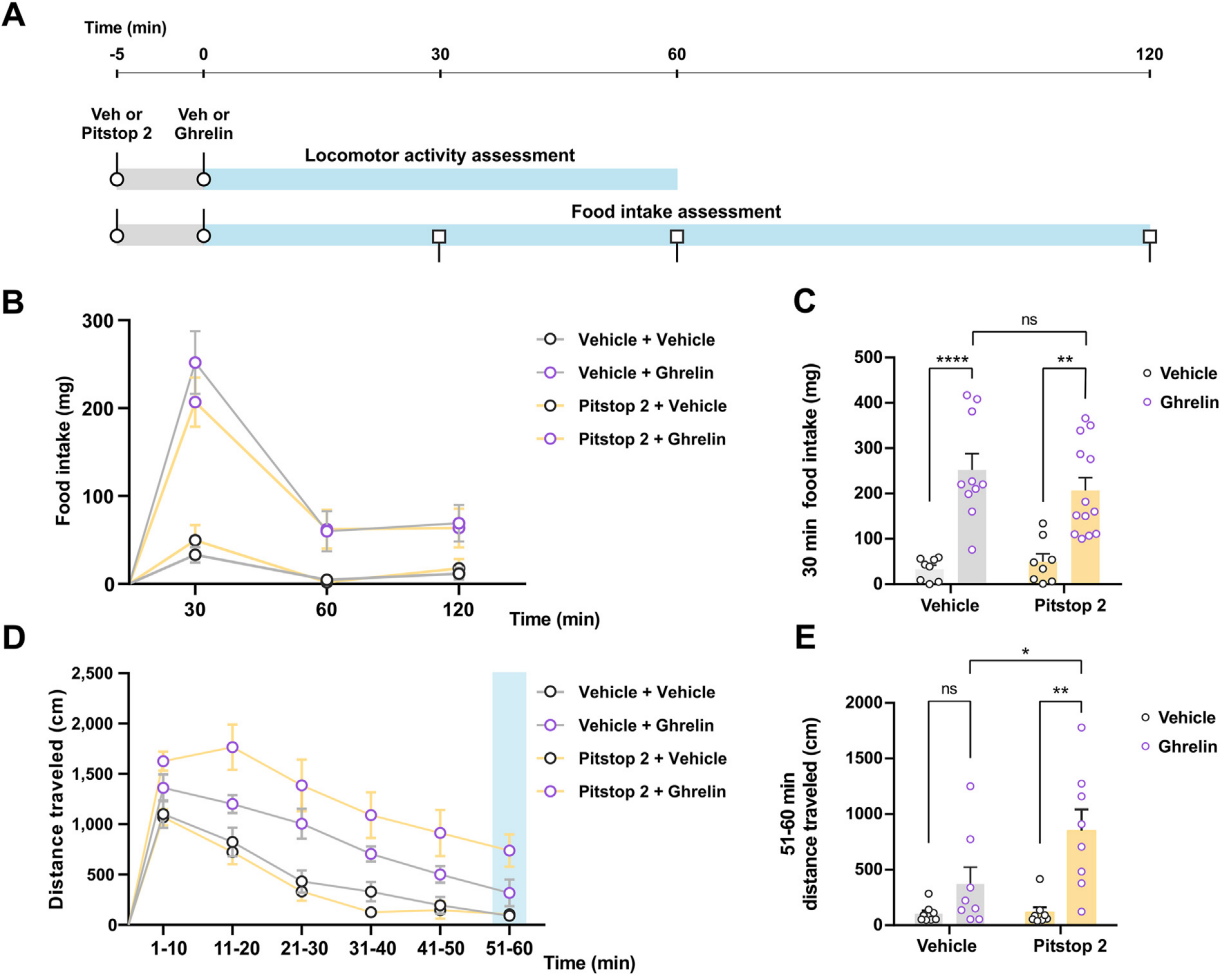
**The internalization of ghrelin from the CSF has functional implications**. Panel **A** shows the experimental outline used in the behavioral assessments. Mice were centrally injected with vehicle or Pitstop 2 and after 5 min injected with vehicle or ghrelin, as appropriate. Food intake was monitored for 120 min or locomotor activity was registered for 60 min after the injections. Panel **B** shows the food intake (mg) over time (min) of mice subjected to the experimental scheme described in A (vehicle + vehicle: n = 8; vehicle + ghrelin: n = 10; Pitstop 2 + vehicle: n = 8; Pitstop 2 + ghrelin: n = 13). Panel **C** shows the quantitative analysis of 30 min-food intake for the mice mentioned in panel B. Two-way ANOVA (F_pre-treatment_ (1,35) = 0.2546; P = 0.6170; F_post-treatment_ (1,35) = 44.69; P < 0.0001; F_interaction_ (1,35) = 1.191; P = 0.2826). Panel **D** show the distance traveled (cm) over time (min) of mice subjected to the experimental scheme described above (n = 8 for every condition). Panel **E** shows the quantitative analysis of locomotor activity in the 51–60 min period for the mice mentioned in panel d. Two-way ANOVA (F_pre-treatment_ (1,7) = 40.23; P = 0.0004; F_post-treatment_ (1,7) = 8.533; P = 0.0223; F_interaction_ (1,7) = 8.836; P = 0.0207). In every case, multiple comparisons were made using Sidak's post hoc test. ∗∗∗∗P < 0.0001; ∗∗P < 0.01; ∗P < 0.05.

In order to study if clathrin-mediated mechanisms in the brain ventricular system affect the ghrelin in the CSF, we assessed CSF ghrelin concentration in mice that had been centrally pretreated with vehicle or Pitstop 2 and then centrally injected with ghrelin. We found that CSF ghrelin concentration was higher in mice pretreated with Pitstop 2 as compared to mice pretreated with vehicle (1.22 ± 0.19 vs 0.77 ± 0.15 pmol/μL, unpaired t-test P = 0.0698). To assess if the higher concentration of ghrelin found in the CSF of Pitstop 2-treated mice has functional consequences, we compared the orexigenic and locomotor effects of centrally injected ghrelin in mice that had been pretreated with Pitstop 2 or vehicle (Figure 6A). We found that central administration of Pitstop 2 did not affect basal food intake nor the orexigenic effect induced by centrally injected ghrelin (Figure 6B–C). Conversely, central administration of Pitstop 2 alone did not affect locomotor activity but significantly increased the effect of central administration of ghrelin on locomotor activity in the 50-to-60 min time period (Figure 6D–E).

## 4 Discussion

Understanding how circulating hormones target the brain to induce physiological responses, including behavioral effects, is a fundamental problem in neuroendocrinology. Currently, very little is known about the molecular mechanisms transporting key peptides to and from the brain. Studies carried out to date on different peptide hormones have provided some controversial and even counterintuitive results, as seen with ghrelin. In this regard, the seminal work by Banks and colleagues in mice showed that systemically-injected radioactive mouse ghrelin displayed very low uptake by the brain whereas centrally injected radioactive ghrelin was rapidly transported out of the brain by a saturable process at a rate that exceeded rates due to the reabsorption of CSF [8]. Studies using fluorescent versions of ghrelin indicated that the low uptake of circulating ghrelin by the brain is presumably due to the fact that this hormone mainly accesses the brain through passive extravasation, which is restricted to the circumventricular organs, and to a lesser extent, through crossing the blood-CSF barrier in the choroid plexus. The observation that ghrelin is rapidly transported in a brain-to-blood direction was rather disregarded, presumably because it was unexpected for a hormone not produced in the brain [40]. Here, we provide the first comprehensive evidence that hypothalamic tanycytes are the cellular entities that mediate the rapid transport of ghrelin out of the brain. We propose that this mechanism has functional implications in controlling the duration of the central effects of ghrelin, as reported here for locomotor activity. Thus, this study not only clarifies the neurobiological basis controlling ghrelin actions in the brain but also unmasks a novel role for hypothalamic tanycytes that could be targeted to manipulate the duration of the central effects of ghrelin for therapeutic goals.

Here, we first investigated the mechanisms mediating the internalization of ghrelin in cultured hypothalamic tanycytes using a unique *in vitro* system that allows us to culture them with their *in vivo* morphology and precisely assess fluorescent signals in the different cell compartments of individual cells [35]. We found that tanycytes rapidly internalize ghrelin via a mechanism that was unaffected by JMV2959, a GHSR ligand that interacts with the ghrelin-binding pocket of the receptor [34]. Thus, tanycytes seem to internalize ghrelin via a selective mechanism that does not require GHSR, in line with our previous studies showing that GHSR mRNA is undetectable in mouse hypothalamic tanycytes and that hypothalamic tanycytes of GHSR-deficient mice actively internalize ghrelin *in vivo* and *in vitro* [17]. We also found that ghrelin internalization in tanycytes was unaffected by scr-F-ghrelin but reduced by native ghrelin or desacyl-ghrelin. Thus, the GHSR-independent mechanism mediating ghrelin internalization in tanycytes seems to depend on the peptide sequence of Fr-ghrelin, rather than on the presence of the acyl group. *In vitro*, ghrelin internalization in tanycytes does not seem to require any carrier protein since our studies were performed in HBSS medium, in the absence of serum or albumin. Notably, we have previously found that hypothalamic tanycytes also rapidly internalize a centrally injected 18-residue analog of desacyl-ghrelin conjugated to fluorescein at its C-terminus *in vivo* [41]. Thus, tanycytes may express a specific receptor that binds to the N-terminal residues shared by ghrelin and desacyl-ghrelin, but this possibility remains speculative, as the existence of a specific receptor for desacyl-ghrelin has not been demonstrated [41,42]. Since ghrelin, Fr-ghrelin and desacyl-ghrelin all bear a positive net charge, it can also be hypothesized that their internalization in tanycytes depends on electrostatic interactions, which can mediate peptide internalization by other endocytic cell types [43]. Further studies are required to determine the specific molecular systems that interact with ghrelin to trigger its internalization.

Current results indicate that ghrelin internalization in hypothalamic tanycytes involves a clathrin-dependent mechanism. *In vitro*, we found that internalized Fr-ghrelin co-localize with clathrin, in line with previous observations [24], as well as with Rab5a and TfR, which are markers of the early endosomes [44] or early and recycling endosomes [45], respectively. Rab5 is involved in the internalization and trafficking of other G protein-coupled receptors by regulating vesicle fusion and receptor sorting in early endosomes [46], suggesting that ghrelin internalization in tanycytes may be mediated by a specific receptor. TfR is not only present in early, clathrin-dependent endosomes but also in endocytic sorting and recycling processes [47], which is compatible with the possibility that internalized Fr-ghrelin may be trafficked through the tanycytes and further exocytosed. Since fluorescent microspheres are non-specifically internalized via clathrin-mediated endocytosis [[48], [49], [50]], the joint internalization of these tracers also suggest that ghrelin is internalized via a clathrin dependent mechanism. Conversely, the observation that F-ghrelin is internalized into different endocytic compartments than scr-F-ghrelin, which does not affect F-ghrelin internalization, suggests that tanycytes utilize distinct endocytic pathways. We also found that ghrelin internalization in cultured tanycytes is reduced by Pitstop 2, which interferes with the clathrin-coated vesicle assembly [30], and Dyngo-4a, which inhibits dynamin action and prevents membrane fission during clathrin-mediated endocytosis [31,32]. The internalization in tanycytes of centrally injected ghrelin was also reduced in Pitstop 2-treated mice. Together, these results support the notion that the internalization of ghrelin mainly depends on a clathrin-dependent mechanism, although other endocytic mechanisms may also play a secondary role. Since clathrin-coated membranes are negatively charged [51], electrostatic interaction could also favor the preferential binding and internalization of the ghrelin; however, clathrin-mediated endocytosis is commonly considered a form of receptor-mediated endocytosis [[51], [52], [53]].

Our results suggest that ghrelin is internalized in the apical pole of tanycytes and then transported to the terminal end. *In vitro*, our pulse-chase and steady state experiments with colchicine, which blocks intracellular transport [33], strongly indicate that Fr-ghrelin is internalized in the soma and then transported to the endfoot. To evaluate the independent capacity of the apical or basal poles of tanycytes to internalize Fr-ghrelin within a conserved structural context, we devised a novel experimental approach. This strategy involves *ex vivo* incubation of horizontally sectioned ventral blocks of the mouse hypothalamus in a two-compartment chamber that maintains the compartmentalization between the intra-ventricular surface and the external ME surface. The use of Fluorogold in this experimental approach indicated that tanycytes are able to internalize cargo molecules from both apical and basal poles, and confirms that the tracer added in the external incubation media access to the extracellular fluid around the ME blood vessels. Conversely, tanycytes were able to internalize Fr-ghrelin only when the tracer was added in the ventricular compartment, but not when the tracer was added in the medium contacting the external ME surface. Thus, tanycytes also seem to internalize Fr-ghrelin from its apical pole in their natural context. Of note, tanycytes have been shown to internalize other molecules from the CSF, such as wheat-germ lectin, ferritin or horseradish peroxidase, and vectorially transport them in a soma-to-endfoot direction [21,54]. The capability of tanycytes to transport molecules in soma-to-endfoot direction is supported by the finding that, in ME coronal sections, clathrin is located in the apical side of the somas and the proximal segment of the processes, but not in the terminal ends of β tanycytes [21].

Hypothalamic tanycytes in rodents can be broadly classified as α or β type depending on their location, spatial relationships, morphology, cytochemistry, ultrastructure and functions [[55], [56], [57]]. Our *in vivo* and *ex vivo* studies showed that the tanycytes that primarily internalize Fr-ghrelin line the floor of the third ventricle indicating that they belong to the β type. Notably, β tanycytes that internalize centrally injected Fr-ghrelin also internalize centrally injected scr-F-ghrelin or fluorescent microspheres indicating their high tendency for internalizing molecules present in the CSF. Interestingly, *in vitro* and *in vivo* experiments showed that Fr-ghrelin rapidly disappears from tanycytes after internalization, suggesting that they are not able to store this peptide hormone. Since the basal processes of β tanycytes project to the external zone of the ME, where the fenestrated capillaries of the pituitary portal vasculature are located, we initially hypothesized that Fr-ghrelin secreted from β tanycytes could act on the pituitary. However, we found no evidence of the presence of Fr-ghrelin in the pituitary of mice that had been centrally injected with the tracer and euthanized at different times after injection (data not shown). Thus, we lack evidence to infer whether Fr-ghrelin is secreted intact by the tanycytes or if it retains the ability to act on pituitary cells. In contrast, all our experiments do strongly indicate that tanycytes play a major role in selectively clearing ghrelin from the CSF. Noteworthy, fluorescent ghrelin is detected in the tanycytes of mice systemically injected with the tracer [16,18]. In light of the current findings, we propose that this observation stems from circulating fluorescent ghrelin initially accessing the CSF through the choroid plexus and then being rapidly internalized by tanycytes, as demonstrated here.

We finally explored if the clathrin-dependent clearance of ghrelin from CSF has any influence on the central effects of ghrelin. Since central Pitstop 2 administration in mice leads to a reduction of Fr-ghrelin internalization in the cell bodies of the tanycytes, as well as in the epithelial cells of the choroid plexus, we hypothesize that pharmacological inhibition of clathrin-mediated endocytosis would lead to a sustained elevation of CSF ghrelin levels, after ghrelin treatment, and consequently in a prolonged duration of the central effects of ghrelin. For food intake, we found that Pitstop 2 did not affect basal or ghrelin-induced food intake. This observation is in line with previous studies showing that systemic ghrelin treatment, which transiently increases plasma ghrelin concentration for ∼40 min, induces a sustained activation of the agouti-related protein-producing neurons of the arcuate nucleus that lasts for several hours [58]. Therefore, the duration of the orexigenic effect of ghrelin appears to primarily depend on the initial activation of orexigenic mechanisms and subsequently be independent of the reduction in ghrelin levels. Conversely, the inhibition of clathrin-mediated endocytosis prolongs the effects of ghrelin on locomotor activity, and makes them evident at later time points, when the effects of ghrelin alone on locomotor activity have dissipated. Ghrelin's effects on locomotor activity has been shown to involve activation of the mesolimbic dopamine system, presumably via indirect mechanisms since the ghrelin fails to induce hyperlocomotion in mice with GHSR expression only in dopamine neurons [[59], [60], [61]]. Notably, a single bolus of centrally injected ghrelin in mice, as done in the current study, has been shown to induce a transient dopamine overflow in the nucleus accumbens that lasts for ∼90 min [61]. Therefore, it is possible that the effects of ghrelin on locomotor activity rely more on the increase in ghrelin levels. This functionally distinct feature, compared to the effects of ghrelin on food intake, may have contributed to reveal a functional implication of the reduced clearance of ghrelin from the CSF in Pitstop 2-treated mice. Further studies are needed to pinpoint the neurobiological underpinnings of these complex behaviors regulated by ghrelin.

This study is the first to provide compelling *in vitro* and *ex vivo* evidence that ghrelin is rapidly internalized in the cell soma of hypothalamic tanycytes and vectorially transported to the endfeet. The mouse studies reported here add valuable cellular and molecular information regarding this process *in vivo*; however, they do have some limitations. For instance, the pharmacological approach used to inhibit endocytosis may affect other cell types beyond tanycytes. Future studies employing more specific methods, such as selectively expressing toxins in tanycytes to block transcytosis [62], would be required to allow for a more precise examination of the role of tanycytes in internalizing ghrelin from the CSF. Additionally, since we initially found that cultured tanycytes primarily internalize ghrelin within the cell soma before transporting it to the terminals, all *in vivo* experiments were conducted using central infusions of ghrelin or Fr-ghrelin. As a result, we have thoroughly characterized the exit of ghrelin from the CSF, though the mechanisms by which it is transported from the blood to the CSF still require further investigation. In this regard, it is well established that some actions of ghrelin, such as its rapid orexigenic effects, mainly involve the diffusion of the hormone and subsequent action on brain areas with fenestrated capillaries [13,63], whereas other actions of ghrelin, including locomotor effects, require its transport into the brain in order to reach targets located farther from the circumventricular organs [38]. Based on our current and previous studies [18], we propose that systemically administered ghrelin in mice is first internalized by epithelial cells of the choroid plexus and takes at least 15 min to increase ghrelin levels in the CSF. Subsequently, it is internalized into the somas of hypothalamic tanycytes, which are capable of internalizing fluorescent ghrelin (Fr-ghrelin) from the CSF within a few minutes. The role of tanycytes in mediating the central effects of endogenous plasma ghrelin elevations remains to be investigated.

## 5 Conclusion

Overall, our study provides compelling evidence that ghrelin is internalized in hypothalamic tanycytes via a GHSR-independent and clathrin-mediated endocytosis mechanism, predominantly occurring in the cell soma, and then vectorially transported in a soma-to-endfoot direction. We propose that tanycytes-mediated endocytosis of ghrelin contributes to clearing this peptide hormone from the CSF, thereby potentially controlling the duration of its central effects.

## CRediT authorship contribution statement

**Ivana M. Gomez:** Writing – review & editing, Methodology, Investigation, Conceptualization. **Maia Uriarte:** Writing – review & editing, Investigation, Formal analysis, Conceptualization. **Gimena Fernandez:** Writing – review & editing, Methodology, Investigation. **Franco Barrile:** Methodology, Investigation. **Daniel Castrogiovanni:** Methodology, Investigation. **Sonia Cantel:** Resources, Investigation. **Jean-Alain Fehrentz:** Resources, Investigation. **Pablo N. De Francesco:** Writing – review & editing, Writing – original draft, Supervision, Conceptualization. **Mario Perello:** Writing – review & editing, Writing – original draft, Supervision, Project administration, Funding acquisition, Conceptualization.

## Declaration of competing interest

The authors declare that the research was conducted in the absence of any commercial or financial relationships that could be construed as a potential conflict of interest.

## Data Availability

Data will be made available on request.
